# Supplementary material for: Plasma Lysophosphatidylcholine Levels Correlate with Prognosis and Immunotherapy Response in Squamous Cell Carcinoma
Source: Int J Mol Sci. 2025 Aug 4;26(15):7528. doi: 10.3390/ijms26157528 (PMC12347404; doi:10.3390/ijms26157528)
Supplement: Supplementary file 1 [file ijms-26-07528-s001.zip › ijms-3752271-supplementary.pdf]

Supplementary Figures

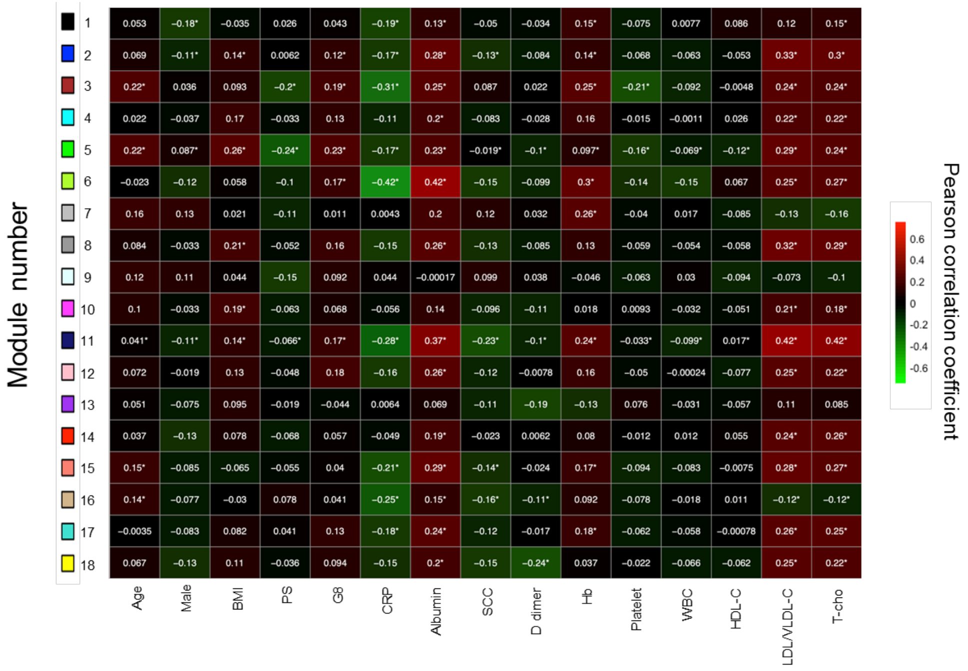

Supplementary figure S1. Heatmap of the Pearson correlation coefficients between the clinical characteristics not shown in figure 2B and module eigengenes derived from the WGCNA. The asterisks (*) indicate the correlations considered significant (q < 0.1). WGCNA, weighted gene correlation network analysis

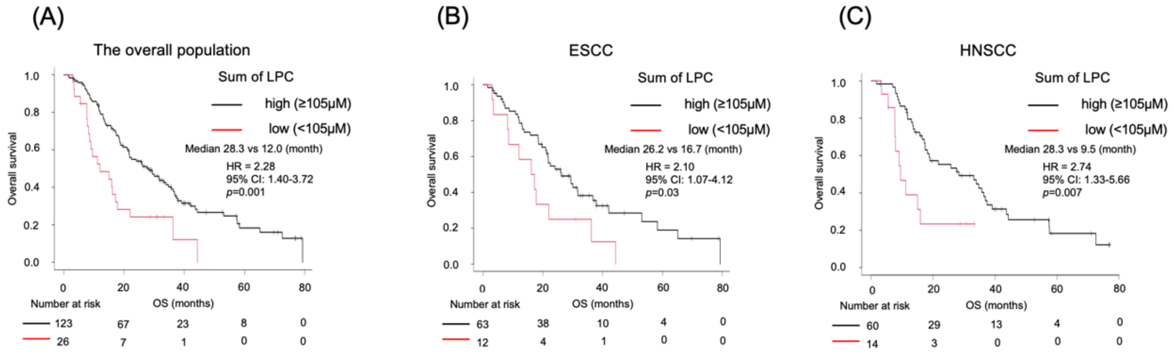

Supplementary figure S2. Kaplan–Meier analysis for OS in different subgroups of patients based on the lower LPC level. (A)–(C) Kaplan–Meier curves of OS between the total LPC

high (≥105 μM) and low (<105 μM) groups in the overall population (A), esophageal SCC group (B), head and neck SCC group (C). LPC, lysophosphatidylcholine; OS, overall survival

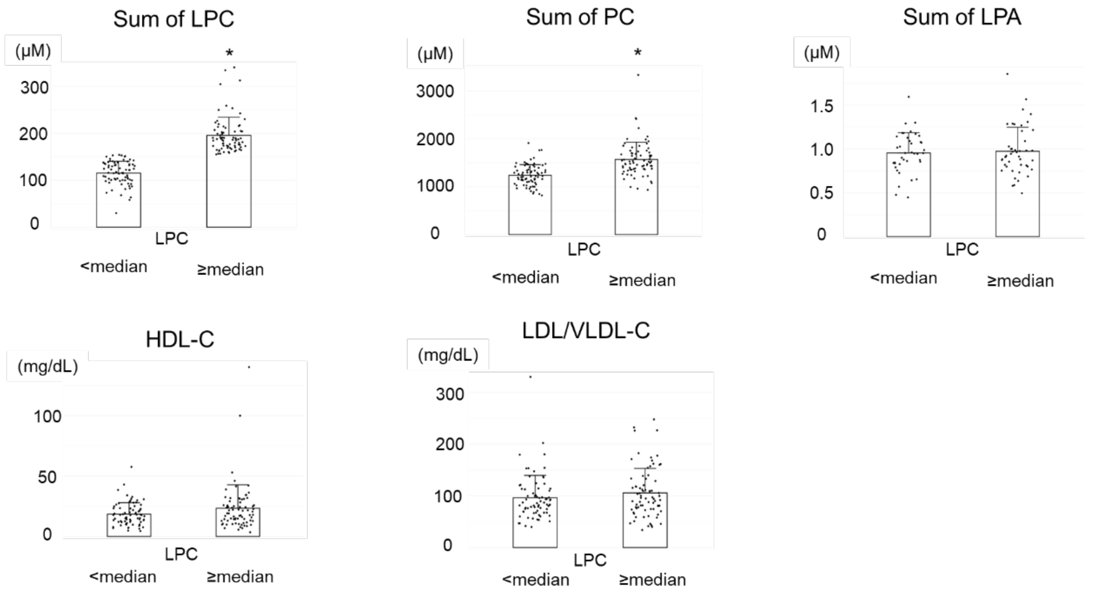

Supplementary figure S3. Comparison of LPC metabolic pathways between total LPC high (≥median) and low (<median) group. PC, phosphatidylcholine; differentially expressed genes; LPC, lysophosphatidylcholine; LPA, lysophosphatidic acid; HDL-C, High-density lipoprotein cholesterol; LDL/VLDL-C, Low-density lipoproteins cholesterol/very low-density lipoproteins cholesterol
